# Avoiding never events in orthopaedics theatres: a quality improvement project

**DOI:** 10.1136/bmjoq-2024-002971

**Published:** 2025-01-27

**Authors:** Bhaskar Amarnath Bhavanasi, Shrikant Kulkarni

**Affiliations:** 1 Trauma & Orthopaedics, Sandwell and West Birmingham Hospitals NHS Trust, Birmingham, UK

**Keywords:** Never events, Quality improvement, Surgery

## Abstract

Never events in the operating room are a surgeon’s nightmare, with an incidence rate of 54%. These events are highly stressful for theatre staff and significantly compromise patient safety. The aim of this project is to avoid never events in trauma and orthopaedic theatres by ensuring that theatre staff adhere to the surgical pause and imaging pause protocols through regular audits.

This prospective study was conducted in both trauma and elective orthopaedic theatres. It involved theatre staff members who were not part of the surgical team. The study was designed to take place on random days across different theatres, with the operating team unaware of the audit to ensure genuine behaviour and compliance.

The audits focused on observing whether the surgical and imaging pause protocols were followed correctly. These protocols are critical for verifying patient identity, the surgical site, and the specific procedure and confirming the correct imaging is available and reviewed before proceeding. Data collected and corrective actions were implemented when non-compliance was observed, and data on compliance rates were systematically collected and analysed.

Preliminary results indicate a substantial increase in compliance with both the surgical and imaging pause protocols, corresponding with a reduction in the occurrence of never events. Theatre staff reported improved understanding and confidence in performing these safety checks The use of external auditors who were not part of the surgical team provided an unbiased assessment of compliance, enhancing the reliability of the findings.

In conclusion, the project demonstrates that regular audits, and data collected by non-surgical team staff, significantly improve adherence to surgical and imaging pause protocols, thereby reducing the incidence of never events in trauma and orthopaedic theatres. This approach highlights the importance of continuous monitoring and education in fostering a culture of safety and precision in surgical practice.

**WHAT IS ALREADY KNOWN ON THIS TOPIC**

- Apart from one recent case series, in surgical specialty, there is a notable lack of detailed literature specifically addressing the prevention of never events in orthopaedic theatres. highlighting a significant opportunity for further research and focused quality improvement initiatives.

**WHAT THIS STUDY ADDS**

- By implementing these targeted strategies, orthopaedic theatres can significantly reduce the risk of never events and improve overall patient safety.

**HOW THIS STUDY MIGHT AFFECT RESEARCH, PRACTICE OR POLICY**

- This study has the potential to inform future research, improve clinical practice and influence healthcare interventions aimed at reducing what should never be involved in orthopaedic surgery.

## Problem

 In our organisation, two never events occurred within a span of 4 months in trauma theatres, highlighting critical lapses in surgical safety protocols.

The first incident involved a woman in her early 70s admitted with a comminuted fracture of her left humerus following a fall at home. She underwent an open reduction and internal fixation of the left upper arm, a procedure involving image intensification (X-ray). The fracture was fixed using a metal plate and screws, with a jig temporarily placed on the plate to assist in screw placement. However, the jig was inadvertently left in place and was only identified the following day during a check X-ray.^1^

The second incident involved a man in his early 60s, who sustained a wrist injury that was internally fixed with metalwork. Due to subsequent pain and restricted movement, he was scheduled for a follow-up operation to remove the metalwork from his right wrist. The right wrist was marked with an arrow indicating the surgical site. The operation commenced with an incision on the thumb side of the wrist, but the scrub nurse questioned this. On reviewing the X-ray, it was discovered that the K-wire to be removed was actually on the little finger side of the wrist.^2^ Raising urgent concerns about our current safety protocols. This prompted the initiation of a focused project to address and mitigate these events.

The primary aim of our project is to reduce the incidence of never events in trauma and orthopaedic theatres by ensuring strict adherence to the surgical pause and imaging pause protocols. Our SMART aim is to decrease the occurrence of never events to below 0% within 12 months across all trauma and orthopaedic theatres in our institution.

Sandwell and West Birmingham NHS Trust is an integrated care organisation renowned for its dedication to training, education, research and innovation. Serving a population of 530 000 people from North-West Birmingham and the towns within Sandwell, the Trust employs over 7000 staff members. City Hospital, a key facility within the Trust, is a 304-bed site located in the heart of Birmingham. It houses several specialised units, including the West Midlands Poisons Unit, Birmingham and Midland Eye Centre, Pan-Birmingham Gynae-Oncology Centre, Sickle Cell and Thalassemia Centre, and Behçet’s National Centres.

## Background

Never events in the NHS are defined as ‘patient safety incidents that are wholly preventable where guidance or safety recommendations that provide strong systemic protective barriers are available at a national level and have been implemented by healthcare providers’.^3^ Despite the fact that never events should never occur, they continue to happen, particularly in surgical settings. The persistence of surgical never events underscores the critical need for effective safety protocols and rigorous adherence to established guidelines.

The Healthcare Safety Investigation Branch has been actively supporting NHS organisations since 2015 by offering guidance on investigations and conducting specific investigations itself. This initiative aims to improve patient safety by providing insights into the root causes of never events and recommending systemic changes to prevent their recurrence.^4^

The Care Quality Commission has identified early emerging themes for preventing never events, emphasising the high-risk nature of healthcare and the necessity of prioritising safety protocols and policies. Acknowledging the inherent dangers in healthcare delivery is essential for mitigating risks and improving patient outcomes. Embedding safety into every aspect of healthcare practice, from decision-making to daily workflows, is crucial for creating a culture of safety. Comprehensive training programmes that highlight the importance of following safety protocols can significantly contribute to this cultural shift.

Healthcare organisations must adopt a proactive approach to risk management by conducting regular risk assessments, implementing quality improvement initiatives and fostering a culture of continuous learning. Robust incident reporting systems are also vital for capturing and analysing errors, near misses and adverse events. Learning from these incidents allows healthcare organisations to identify areas for improvement and implement changes to prevent similar occurrences in the future.^5^

The introduction of the WHO Surgical Safety Checklist in 2010 was a significant step towards enhancing patient safety during invasive procedures. This checklist was designed to ensure that healthcare teams adhered to a standardised set of safety checks before, during and after surgeries, thereby reducing the incidence of never events such as wrong-site surgery. However, despite the implementation of the WHO Surgical Safety Checklist, a substantial reduction in never events was not observed.

In response to the continued occurrence of never events, NHS England launched the National Safety Standards for Invasive Procedures (NatSSIPs) in 2015. NatSSIPs aimed to establish national standards for invasive procedures and provide guidance for healthcare organisations to develop their own Local Safety Standards for Invasive Procedures (LocSSIPs). This approach was intended to harmonise clinical practice and ensure consistency in safety protocols across the NHS.

By implementing NatSSIPs and encouraging the development of LocSSIPs, healthcare organisations can tailor safety standards to their specific contexts while adhering to national guidelines. This standardisation of clinical practice is designed to improve patient safety and reduce the occurrence of never events associated with invasive procedures, thereby enhancing the overall quality of care within the NHS.^6^

In summary, while significant efforts have been made to address never events in the NHS through initiatives like the WHO Surgical Safety Checklist and NatSSIPs, challenges remain. Continuous education, proactive risk management and a culture of safety are essential for further reducing the incidence of these preventable incidents and ensuring the highest standards of patient care.

## Measurement

Measured number of ‘never events’ in Trauma and Orthopaedics (T&O). Never events are serious, largely preventable patient safety incidents that should not occur if the available preventive measures are implemented. Tracking the occurrence of never events directly measures the safety and quality of surgical procedures. Never events in this context refer specifically to incidents where incorrect implants are used during surgical procedures, retained objects or wrong side operations in T&O. This measure is highly valid as it directly relates to the safety objective of the intervention. It is reliable as the data is collected and published annually by the Trust, ensuring standardised reporting and consistency over time.

Data were collected from Trust Never Events publications, which are publicly available and released annually.

Baseline measurement:

- 2013/2014: 1 case reported.^7^ (Wrong implant in late 2013 during a total hip replacement operation.)
- 2014/2015: 0 cases.
- 2016/2017: 2 cases.
- 2018/2019: 0 cases.
- 2019/2020: 0 cases.
- 2020/2021: 0 cases.
- 2021/2022: 0 cases.
- 2022/2023: 0 cases.

The baseline measurement spans several years, allowing for a comparison before and after the intervention.

Case 1—11 years ago—wrong sized implant was used while performing total hip replacement.

Case 2—7 years ago—a zig has been left along with the plate while fixing the humeral fracture.

Case 3—7 years ago—the wrong side of the arm has been operated on to remove a K wire.

### Intervention

Implementation of the surgical pause protocol in theatres, specifically within T&O departments. Surgical pause protocols are designed to prevent errors by ensuring that surgical teams confirm the correct patient, procedure, and implant before proceeding. Data will be collected annually, in line with the Trust’s publication schedule.

By comparing the frequency of never events before and after the implementation of the surgical pause protocol, we can establish if the observed outcomes (ie, reduction in never events) are due to the intervention. The clear distinction in the data, with never events ceasing from 2018 onwards, supports this causal relationship. This data shows a noticeable drop in never events following the implementation of the surgical pause protocol in 2018.

The data prior to 2018 shows occasional never events, while the absence of never events from 2018 onwards suggests a successful intervention.

The chosen measure (number of never events) is a valid and reliable indicator of the intervention’s success. The baseline data and the continued absence of never events postintervention strongly suggest that the surgical pause protocol has been effective in improving patient safety in T&O departments. The data collection plan, focusing on annual publication reviews, ensures consistency and allows for ongoing monitoring of the intervention’s impact.

## Design

To enhance the quality of care in T&O, a series of planned interventions were implemented, driven by immediate responses to specific cases and ongoing efforts to prevent recurrence and enhance safety. These interventions were developed through discussions within the Surgery A Clinical Group, involving surgeons, scrub nurses, anaesthetists, administrative staff and suppliers for instrument tray standardisation. The consultation process included division meetings with the project team and collaboration with suppliers to standardise instrument trays and implement colour differentiation. Engagement methods involved regular meetings, collaborative decision-making and training sessions to ensure all team members understood and could implement the new procedures effectively.

Following incidents such as the wrong implant 11 years ago and a forgotten jig, several measures were put in place. For all implant procedures, a surgical pause was introduced where the operating team stopped to check the implants for side, size and expiry date, and recorded any guides and jigs on the theatre whiteboard. For multiple bone operations with implants to be removed, an imaging pause was implemented, requiring team members to stop and read the X-ray for side and site information, ensuring the correct patient and the absence of retained objects in the operative site.

Further preventive measures included having scrub nurses visually confirm each item during the instrument count, standardising relevant instrument trays, adding risks to the Theatres Risk Register, assessing the feasibility of making the drill guide a different colour from surgical plates and improving the patient transfer process by not sending for the next patient until the final count is completed.

The rationale behind these interventions includes ensuring the correct implant and procedure through surgical pauses, confirming X-ray information through imaging pauses, and improving instrument counting accuracy through visual confirmation and colour differentiation. These practices aim to reduce errors related to patient misidentification, incorrect procedures and retained surgical items, with the assumption that these interventions would significantly reduce never events. Engaging the surgical team and standardising practices are expected to enhance compliance and consistency in safety procedures.

Anticipated problems include resistance to change, logistical challenges in standardising instrument trays and ensuring consistent compliance with new protocols. Mitigation strategies involve continuous engagement and communication with the surgical team, step-by-step implementation of changes with ongoing monitoring and support, and feedback loops to promptly identify and resolve issues.

To sustain these interventions, new procedures will be integrated into standard operating protocols, regular audits will be conducted to monitor compliance and identify improvement areas, ongoing training will be provided to maintain awareness and adherence, and feedback mechanisms will be established for continuous staff input and refinement of interventions.

## Strategy

Our strategy for improvement involved the implementation of continuous, iterative Plan-Do-Study-Act (PDSA) cycles to systematically test and refine our approach to reducing never events in the operating theatre. We aimed to achieve a 0% rate of never events within 12 months. Each PDSA cycle focused on specific aspects of the surgical and implant pause processes, ensuring that changes were evidence-based and grounded in real-time data collection.

### PDSA cycle 1

The aim is to assess the current implementation rate of surgical pause and imaging pause practices.

Data were collected randomly by nursing staff from February 2017 to August 2017. The nursing staff were not part of the operating team, and the operating teams were unaware of the data collection to ensure unbiased results.

Key learning is that the audit revealed that while all teams observed the required pauses, there was uncertainty about the specific timing for observing imaging pauses.

Achieved a 100% implementation rate for both pauses, indicating initial success but highlighting the need for clear guidelines.

Impact on change process, we clarified that imaging pauses should be observed soon after the WHO checklist for patients with intended implant removal, and surgical pauses should occur just before opening implant packs and again before wound closure. Presented the findings at the QIHD meeting in December 2017. The changes were explained in the theatre staff meeting which takes place every Tuesday at 08.00. Developed and displayed posters in the theatres to clarify when to observe imaging and surgical pauses.

### PDSA cycle 2

The aim is to verify the sustainability of 100% implementation of surgical and imaging pauses and to test if the clarifications made in cycle 1 were effective.

Data were collected by ODPs, anaesthesia registrars and consultants from June 2021 to December 2021, with operating teams unaware of the data collection. Data collection by various roles within the team to ensure comprehensive monitoring. Results were presented and discussed at the QIHD meeting in January 2022.

Consistent 100% implementation was maintained, demonstrating the effectiveness of the changes made in cycle 1. Reaffirmed the effectiveness of the changes and emphasised the importance of continuous monitoring.

The decision was made to conduct regular audits to ensure ongoing adherence and sustainability.

### PDSA cycle 3

Aimed to ensure the continued sustainability of 100% implementation of surgical and imaging pauses and to integrate these practices into the induction of new staff.

Integrating surgical and imaging pause protocols into staff induction and regular audits will sustain 100% implementation and ensure new staff are aware of the protocols.

Data were collected by trauma coordinators from February 2023 to September 2023. Operating teams remained unaware of the data collection. Results were presented and discussed at the December 2023 QIHD meeting. Maintained 100% adherence, indicating robust sustainability.

Established a sustainable practice with ongoing 100% adherence and ensured all staff, including new members, are consistently aware of and adhere to the protocols.

Decided to include surgical and imaging pause protocols in the induction process for new staff and to continue regular audits to maintain high standards.

**Figure 1:**
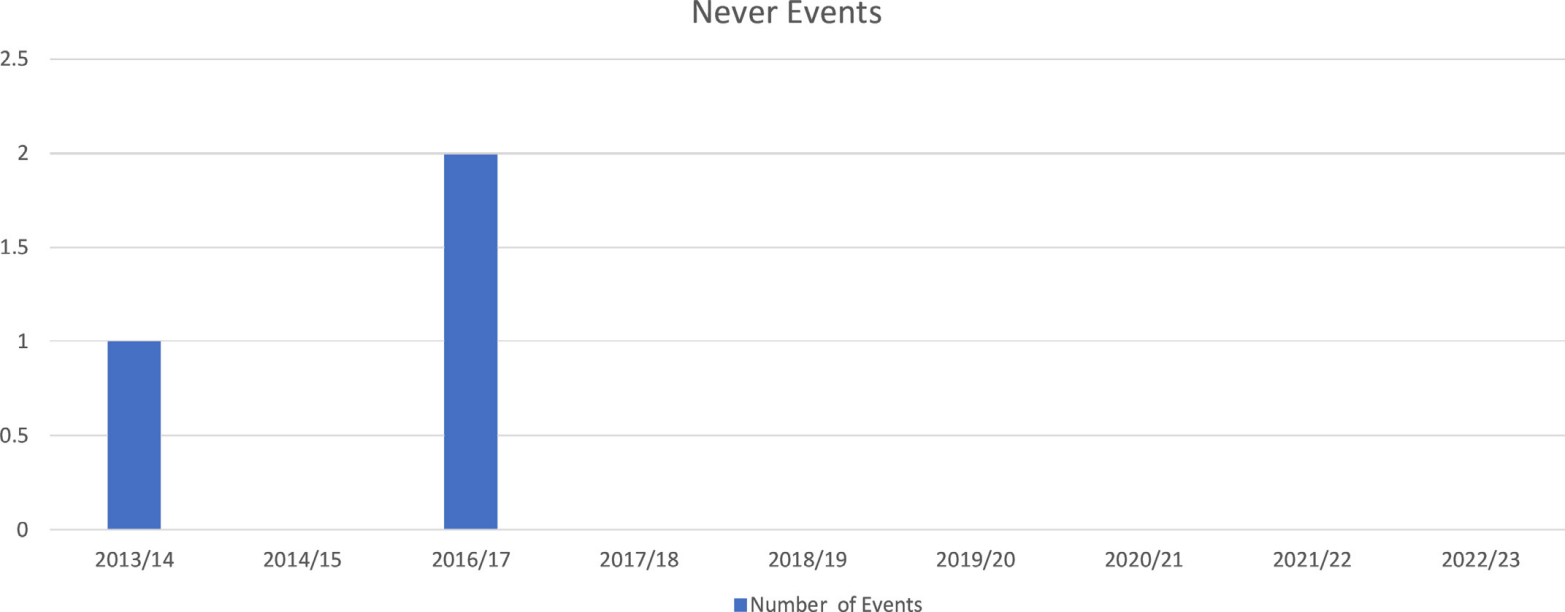
Incidence of never events.

As 100% implementation of surgical and implant, pause resulted in 0 never events (figure 1).

Although no formal education sessions were conducted, the topic was discussed during weekly theatre staff meetings, as noted previously. To reinforce awareness and adherence to protocols, posters were printed and prominently displayed in the theatres, positioned next to the WHO checklist. Staff members are required to read each line of the checklist prior to the commencement of every operation, ensuring consistent attention to safety protocols

**Figure 2:**
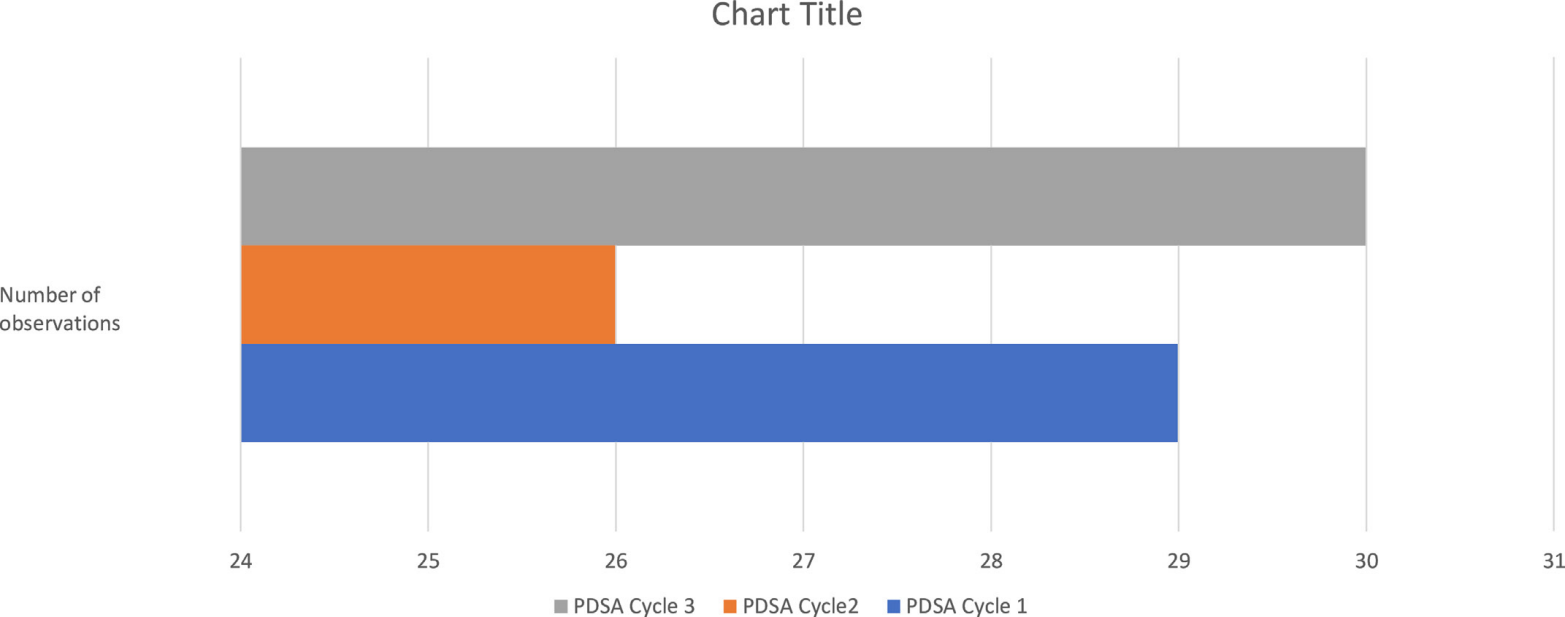
Number of observations in each cycle. PDSA, Plan-Do-Study-Act.

Data were gathered by theatre staff, specifically nursing staff, using a structured data collection tool. Cases for data collection were selected at the discretion of the staff members. Data were collected for 29 theatre days and analysed (figure 2).

## Results

**Figure 3:**
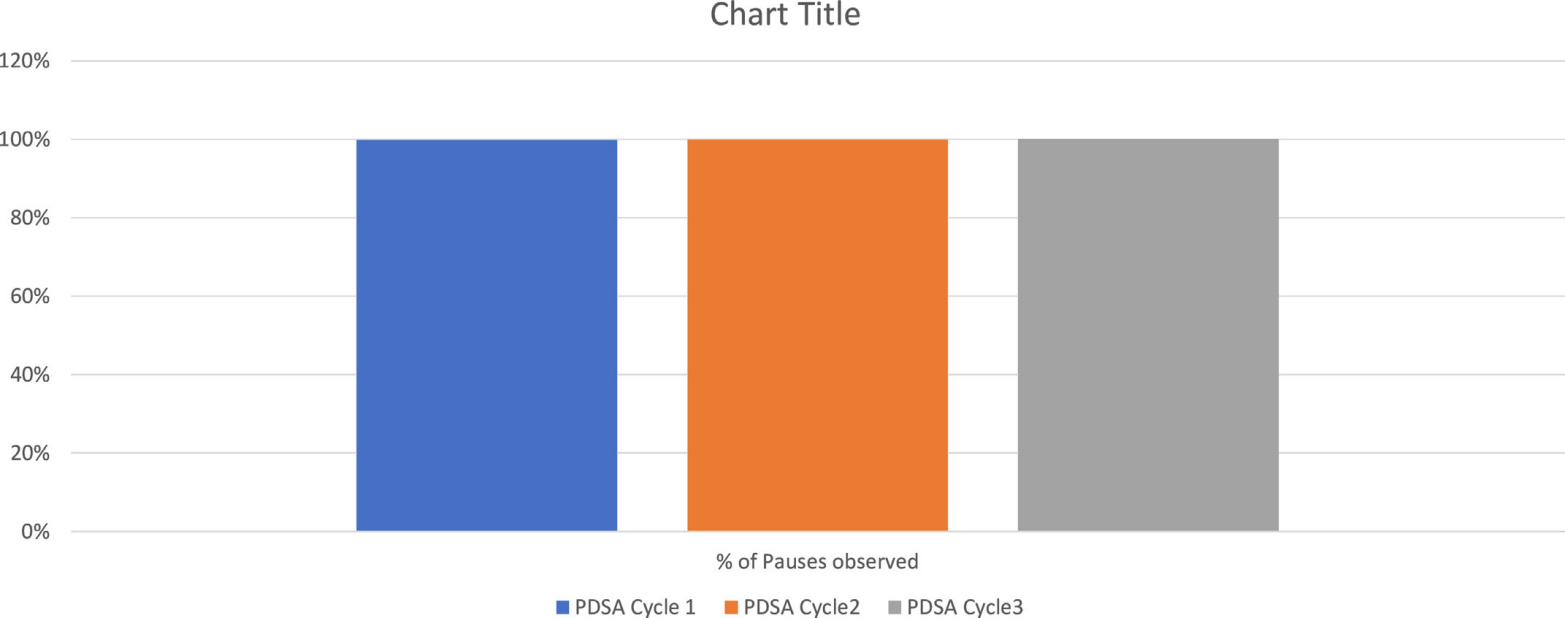
Pauses observed percentage. PDSA, Plan-Do-Study-Act.

Our data indicate zero never events since implementing surgical and imaging pauses from 2017 to 2023, compared with one incident in 2013 and two in 2016. This substantial reduction suggests these interventions have been effective in achieving and sustaining the SMART target of zero never events. Data were collected in real-time and at random over several months per cycle, with operating teams unaware of the process. Despite challenges due to staff schedules and the need for observer presence during pauses, the same data collection tool was used across all cycles, reinforcing the reliability of our findings and providing strong evidence that these interventions are successful in eliminating never events in a surgical setting (figure 3).

Online supplemental file 1 explains the data collection tool.

### Lessons learnt and limitations

A key lesson from our project is the importance of interdepartmental collaboration. Initially, we faced resistance from surgical teams due to the additional workload required to implement surgical and imaging pauses. However, through repeated meetings, teamwork and emphasising patient safety, we gained their cooperation. Finding non-surgical staff to collect data was challenging, but offering certificates of participation from the clinical effectiveness department proved effective in recruiting volunteers. Ensuring data collection involved staff from diverse backgrounds, such as ODP, nursing and anaesthesia consultants, helped maintain the objectivity and reliability of the data. Using a simple and consistent data collection tool across all cycles maintained data integrity and comparability while collecting data randomly and without the surgical teams’ knowledge minimised bias and ensured a representative sample of different teams.

Despite these successes, we encountered several limitations. One significant issue was the difficulty in finding staff members who were not part of the surgical team to collect data due to their busy schedules, making the process time-consuming and challenging. The project could have benefited from a larger sample size.

Reflecting on these lessons and limitations, our project demonstrates significant strengths and areas for future improvement. The implementation of surgical and imaging pauses was effective in achieving and maintaining the SMART target of zero never events. The use of a consistent data collection tool and the involvement of diverse staff members ensured reliable and unbiased data, and the project demonstrated that achieving zero never events is sustainable over an extended period. In future iterations, increasing the sample size by allocating more resources to data collection would provide more robust data. Implementing a continuous feedback loop where results are regularly reviewed and shared with teams could enhance engagement and foster a culture of continuous improvement. Expanding the project to include multiple institutions could improve the generalisability of the findings and provide more comprehensive data. Providing regular training and updates for all staff on the importance and impact of surgical and imaging pauses could maintain high compliance and awareness.

## Conclusion

The persistence of never events in surgical settings underscores the critical need for effective safety protocols and rigorous adherence to established guidelines. Our project aimed to reduce the incidence of never events in trauma and orthopaedic theatres by ensuring strict adherence to surgical and imaging pause protocols through regular audits. This project has successfully demonstrated that regular audits, when combined with data collection by non-surgical team staff, significantly improve adherence to these protocols, thereby reducing the incidence of never events. The implementation of these measures has resulted in a sustained period without never events, highlighting the effectiveness and sustainability of our interventions.

Reflecting on our initial aims, the project has successfully achieved and maintained the SMART target of zero never events. The use of a consistent data collection tool and the involvement of diverse staff members ensured reliable and unbiased data, reinforcing the validity of our findings. Despite initial resistance and logistical challenges, interdepartmental collaboration and the incentivisation of participation played crucial roles in the project’s success. The lessons learnt from this project, including the importance of clear communication, teamwork and continuous monitoring, provide valuable insights for other institutions aiming to improve patient safety. Additionally, our project demonstrated potential financial savings by preventing the costly and complex consequences of never events, though a detailed cost analysis was beyond the scope of this report.

Sustaining the project’s success involves continuous engagement with staff, regular audits and integrating safety protocols into the induction process for new employees. Regular training and updates on the importance of surgical and imaging pauses are essential for maintaining high compliance and awareness.

To prevent never events technology can be used, artificial intelligence (AI) can greatly enhance safety in operating theatres and help prevent never events, such as wrong-site surgeries, retained surgical instruments and medication errors. By using the preoperative planning and real-time monitor AI uses computer vision and sensor data to monitor surgeries, detecting deviations from standard procedures and alerting teams to potential errors like incorrect site marking or retained instruments.

AI-enabled decision support systems can enhance patient safety by improving error detection, patient stratification, and drug management, but further validation in real-world clinical settings is needed.^8^

## supplementary material

10.1136/bmjoq-2024-002971 online supplemental file 1

## Data Availability

Data are available in a public, open access repository.
