# Supplementary material for: Avoiding never events in orthopaedics theatres: a quality improvement project
Source: BMJ Open Qual. 2025 Jan 27;14(1):e002971. doi: 10.1136/bmjoq-2024-002971 (PMC11772924; doi:10.1136/bmjoq-2024-002971)
Supplement: online supplemental file 1 [file bmjoq-14-1-s001.pdf]

Sandwell and West Birmingham Hospitals NHS Trust

SURGICAL PAUSE AUDIT
Audit tool

Date	RXK Number	Theatre Number	Consultant in charge
____/____/________	__________________	______________	__________________________

**1.**	Did the team '**announce**' the surgical pause and **stop** the procedure for the surgical pause to take effect?<br>**Comments:**	Yes ☐ No☐<br>Yes ☐ No☐
**2**	Did the team '**announce**' the **x ray (Imaging) pause** and **stop** the procedure for the x ray pause to take effect?<br>**Comments:**	Yes ☐ No☐<br>Yes ☐ No☐
**3.**	Circulator presented implants to scrub nurse and lead surgeon?<br>**Comments:**	Yes ☐ No☐
**4.**	Implants viewed by scrub nurse: Size, Side, Expiry date?<br>**Comments:**	Yes ☐ No☐
**5.**	Implants viewed by surgeon: Size, Side, Expiry date?<br>**Comments:**	Yes ☐ No☐
**6.**	Any discrepancies identified by scrub nurse?<br>**Comments:**	Yes ☐ No☐
**7.**	Any discrepancies identified by surgeon?<br>**Comments:**	Yes ☐ No☐
**8.**	Incorrect implants opened?<br>**Comments:**	Yes ☐ No☐
**9.**	Surgical pause formally closed?<br>**Comments:**	Yes ☐ No☐
**10.**	Incorrect implants implanted?<br>**Comments:**	Yes ☐ No☐
**11.**	Total time for surgical pause?<br>**Comments:**	----------- mins
